# Identification of Novel Antimicrobial Compounds Targeting *Mycobacterium tuberculosis S*-Adenosyl-L-Homocysteine Hydrolase Using Dual Hierarchical In Silico Structure-Based Drug Screening

**DOI:** 10.3390/molecules29061303

**Published:** 2024-03-14

**Authors:** Hazuki Ito, Kohei Monobe, Saya Okubo, Shunsuke Aoki

**Affiliations:** Department of Bioscience and Bioinformatics, Graduate School of Computer Science and Systems Engineering, Kyushu Institute of Technology, Iizuka 820-8502, Japan

**Keywords:** tuberculosis, *Mycobacterium*, *Mycobacterium tuberculosis S*-adenosyl-L-homocysteine hydrolase (MtSAHH), in silico structure-based drug screening (SBDS), docking simulation, molecular dynamics simulation

## Abstract

The emergence of multidrug-resistant and extensively drug-resistant *Mycobacterium tuberculosis* (*M. tuberculosis*) has become a major medical problem. *S*-adenosyl-L-homocysteine hydrolase (MtSAHH) was selected as the target protein for the identification of novel anti-TB drugs. Dual hierarchical in silico Structure-Based Drug Screening was performed using a 3D compound structure library (with over 150 thousand synthetic chemicals) to identify compounds that bind to MtSAHH’s active site. In vitro experiments were conducted to verify whether the nine compounds selected as new drug candidates exhibited growth-inhibitory effects against mycobacteria. Eight of the nine compounds that were predicted by dual hierarchical screening showed growth-inhibitory effects against *Mycobacterium smegmatis* (*M. smegmatis*), a model organism for *M. tuberculosis*. Compound **7** showed the strongest antibacterial activity, with an IC_50_ value of 30.2 µM. Compound **7** did not inhibit the growth of Gram-negative bacteria or exert toxic effects on human cells. Molecular dynamics simulations of 40 ns using the MtSAHH–Compound **7** complex structure suggested that Compound **7** interacts stably with the MtSAHH active site. These in silico and in vitro results suggested that Compound **7** is a promising lead compound for the development of new anti-TB drugs.

## 1. Introduction

Tuberculosis (TB) is an infectious disease caused by *Mycobacterium tuberculosis* (*M. tuberculosis*) entering the human body. TB is primarily treated with medical therapy using drugs, and the disease is cured when patients continuously take multiple drugs at appropriate doses for approximately 6–9 months. Although the disease can be cured with appropriate medication, it is a serious infectious disease worldwide, with an estimated 10.6 million cases and 1.3 million deaths in 2022. The infection is most prevalent in developing countries in Southeast Asia (46%), Africa (23%), and the West Pacific (18%) [1]. The high cost of treating drug-resistant TB and the lack of satisfactory treatment in developing countries are major global problems. *M. tuberculosis* that has acquired resistance to the first-line drugs isoniazid and rifampicin, which are particularly important drugs in the treatment of TB, is called multidrug-resistant TB (MDR-TB), and those that fail to respond to several other existing drugs and show resistance are called extensively drug-resistant TB (XDR-TB), making treatment extremely difficult. As drug-resistant *M. tuberculosis* is spreading at an alarming rate worldwide, there is an urgent need to develop new drugs that are inexpensive, stably supplied in developing countries, and effective against MDR-TB and XDR-TB [2].

In this study, we focused on *M. tuberculosis S*-Adenosyl-L-Homocysteine Hydrolase (MtSAHH) as a target for in silico anti-TB drug development. MtSAHH reversibly hydrolyzes *S*-adenosylhomocysteine (SAH) to adenosine (ADO) and L-homocysteine (HCY). As SAH acts as a feedback inhibitor of many S-adenosylmethionine (SAM)-dependent methyltransferases, it plays an important role in removing coenzyme products and maintaining a proper SAM/SAH equilibrium. Disturbances in this equilibrium inhibit the growth of various cells. MtSAHH is a potential target for anti-TB drug development because of its essential role in the growth of *M. tuberculosis* [3].

The DUD-E dataset was used to validate the optimal combination and order of screening tools to increase the accuracy of the selection of hit compounds during the in silico drug screening process. A screening pathway was constructed by sequentially combining three tools for docking simulation. Using a chemical structural library (154,118 chemicals), nine candidate compounds were identified using Structure-Based Drug Screening (SBDS), which employs a dual hierarchical method of screening. In vitro mycobacterial antibacterial assays of the candidate compounds showed that eight compounds had growth-inhibitory effects against *Mycobacterium smegmatis* (*M. smegmatis*), a model organism for *M. tuberculosis*. One compound showed strong growth-inhibitory effects against *M. smegmatis,* with an IC_50_ value of 30.2 μM. Moreover, molecular dynamics (MDs) calculations using the complex structure consisting of MtSAHH and the most effective compound indicated that the compound stably interacts with the MtSAHH active site.

## 2. Results and Discussion

### 2.1. In Silico SBDS Pathway

Three docking simulators, GOLD [4], AutoDock Vina (ADV) [5], and DOCK [6], were used to establish the simulation pathways [7]. The dataset of DUD-E compounds (active compounds, 544; inactive compounds, 19,831) was validated for the 2P54 protein structure [8]. The Enrichment Factor (EF) for each pathway was calculated to determine the tools used in the in silico drug screening and their order of use [9]. The EF_0.2%_ values for pathways A, B, C, and D were 17.34, 10.97, 14.60, and 6.37, respectively (Figure S1). Interestingly, swapping the tools used in the first and second phases resulted in a significant change in EF values, suggesting that the hierarchical screenings were effectively constructed. Based on these results, we performed in silico SBDS using pathways A and C.

### 2.2. Dual Hierarchical In Silico SBDS of MtSAHH Using Dual Pathways

**Figure 1:**
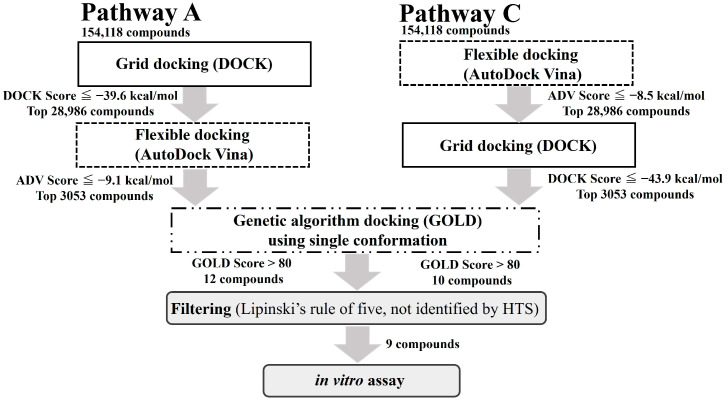
Flowchart of dual hierarchical in silico SBDS.

Dual hierarchical in silico SBDSs were performed with pathways A and C to search for compounds interacting with the MtSAHH active site by employing a 3D chemical structural library (154,118 chemicals) [10] (Figure S2 and Figure 1). Nine candidate compounds were identified for pathways A and C (Table S1). In the first screening using DOCK for pathway A, 28,986 compounds with binding free energy below −39.6 kcal/mol were selected. In the second screening using ADV, we performed binding simulations of these 28,986 compounds and selected 3053 compounds with binding free energy below −9.1 kcal/mol. In the third screening, GOLD selected 12 compounds from the 3053 compounds. The GOLD scores of the 12 compounds were higher than 80. In the first screening using ADV in Pathway C, 28,986 compounds with binding free energy below −8.5 kcal/mol were selected. In the second screening, DOCK selected 3053 compounds from the 28,986 compounds. The binding free energies of the 3053 compounds were below −43.9 kcal/mol. In the third screening, GOLD selected 10 compounds from the 3053 compounds. The GOLD scores of the 10 compounds were higher than 80. The group of compounds selected by pathways A and C consisted of 22 compounds, of which 3 were common (Compound **1**–**3**). The final GOLD scores obtained from Pathway A and Pathway C for these three compounds were all different, even though they were for the same compound. Since GOLD employs a genetic algorithm, it produces minute differences in the GOLD score, even for docking simulations of the same protein and the same compound. In pathway A, GOLD simulations were performed using the compound structure and configuration information of the post-ADV simulation (flexible docking). In pathway C, the GOLD simulation was performed using the configuration information of the post-DOCK simulation (rigid body docking). These differences in initial structure and configuration information that were used in GOLD likely generated the differences in the GOLD score (Table S1). From the remaining uncommon compounds, six compounds that conformed to Lipinski’s law [11] and had not been assayed by high-throughput screening (HTS) [12] were selected for a total of nine compounds in the final selection. Table S1 shows the compounds’ name, ChemBridge ID, IUPAC name, GOLD score, and pathway ID. The small co-assembly of the compound groups selected by the two pathways indicates the uniqueness of the compound selection properties of each pathway.

### 2.3. Verification of Antibacterial Effects of Compounds against M. smegmatis

**Figure 2:**
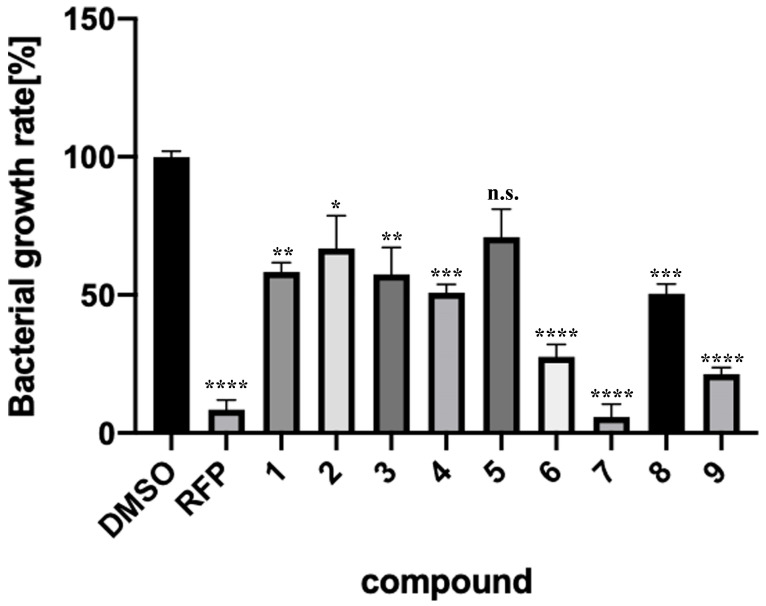
Inhibitory effects of nine compounds (Compounds **1**–**9**) on growth of *M. smegmatis*. Negative control is Dimethyl sulfoxide (DMSO [0.3%]). Positive control is rifampicin (RFP [100 µM]). Final compound concentration = 100 µM. All values were obtained from four independent experiments representing mean ± SEM. Dunnett’s test was performed. *; *p* < 0.0332. **; *p* < 0.0021. ***; *p* < 0.0002. ****; *p* < 0.0001. not significant; n.s.

**Figure 3:**
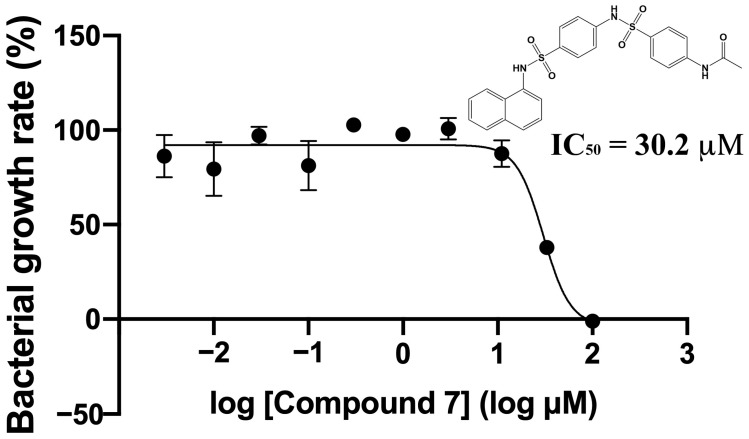
IC_50_ value determination of Compound **7** for *M. smegmatis* growth. Vertical axis: bacterial growth rate (%). Horizontal axis: compound concentration. All values were obtained from four independent experiments representing mean ± SEM. Values of IC_50_ were calculated by Prism 8 software.

Nine compounds were selected by in silico SBDS, and their growth-inhibitory effects on non-pathogenic *M. smegmatis* (biosafety level 1) were tested (Figure 2). Eight of the nine compounds (Compounds **1**, **2**, **3**, **4**, **6**, **7**, **8**, and **9**) blocked the *M. smegmatis* growth. Compound **7** had particularly strong growth-inhibitory activity, with an IC_50_ value of 30.2 μM (Figure 3). The active compound rate was 88.9% (8/9), indicating that the novel method constructed by using dual pathways was remarkably effective.

### 2.4. Toxicity Verification for Escherichia coli and Mammalian Cells

**Figure 4:**
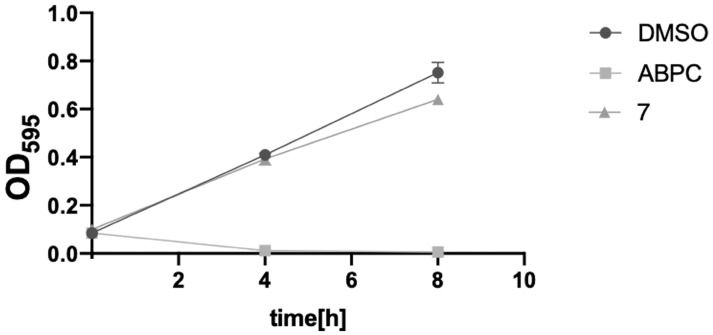
*E. coli* growth-inhibitory effect of Compound **7**. Negative control is ampicillin (ABPC [100 µM]). Positive control is DMSO (0.3%). Final compound concentration = 100 µM. All values were obtained from four independent experiments representing mean ± SEM. Vertical axis represents OD_595_ value of *E. coli,* and horizontal axis represents incubation time.

**Figure 5:**
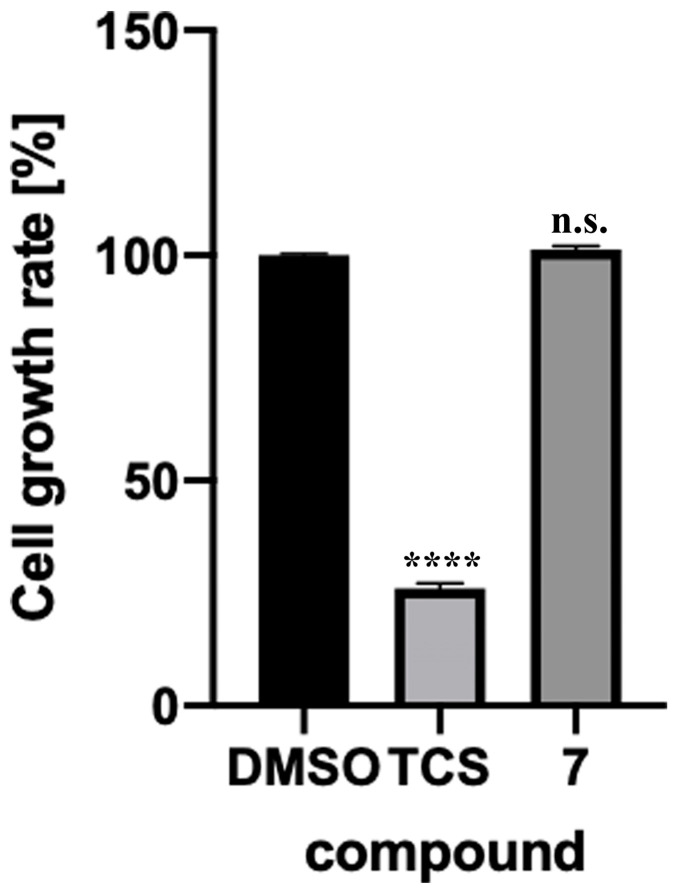
Toxicity verification of Compound **7** on human liver-derived cells. Compound **7** was applied to cultured HepG2 cells. Negative control is triclosan (TCS [50 µM]). Positive control is DMSO (0.3%). Final compound concentration = 50 µM. All values were obtained from four independent experiments representing mean ± SEM. Dunnett’s test was performed. ****; *p* < 0.0001. not significant; n.s.

Since anti-TB drugs must be administered long-term and must not be toxic to intestinal bacteria, we tested the toxicity of Compound **7** against *Escherichia coli* (*E. coli*), a model intestinal bacterium [13]. Compound **7** showed no significant growth inhibition or toxicity against *E. coli* after 4 h or 8 h of incubation (Figure 4). The result suggests that Compound **7** is likely ineffective against Gram-negative bacteria. The toxicity of Compound **7** in cultured human cells was evaluated. Compound **7** showed no significant toxicity against HepG2 cells (human liver-derived cells) (Figure 5). The active center pocket structures of human SAHH and MtSAHH are very similar, making it difficult to identify specific inhibitors of MtSAHH [14]. Compound **7** was expected to bind specifically to and inhibit MtSAHH, but not human SAHH.

### 2.5. Molecular Dynamics Simulation of MtSAHH–Compound ***7*** Complex

**Figure 6:**
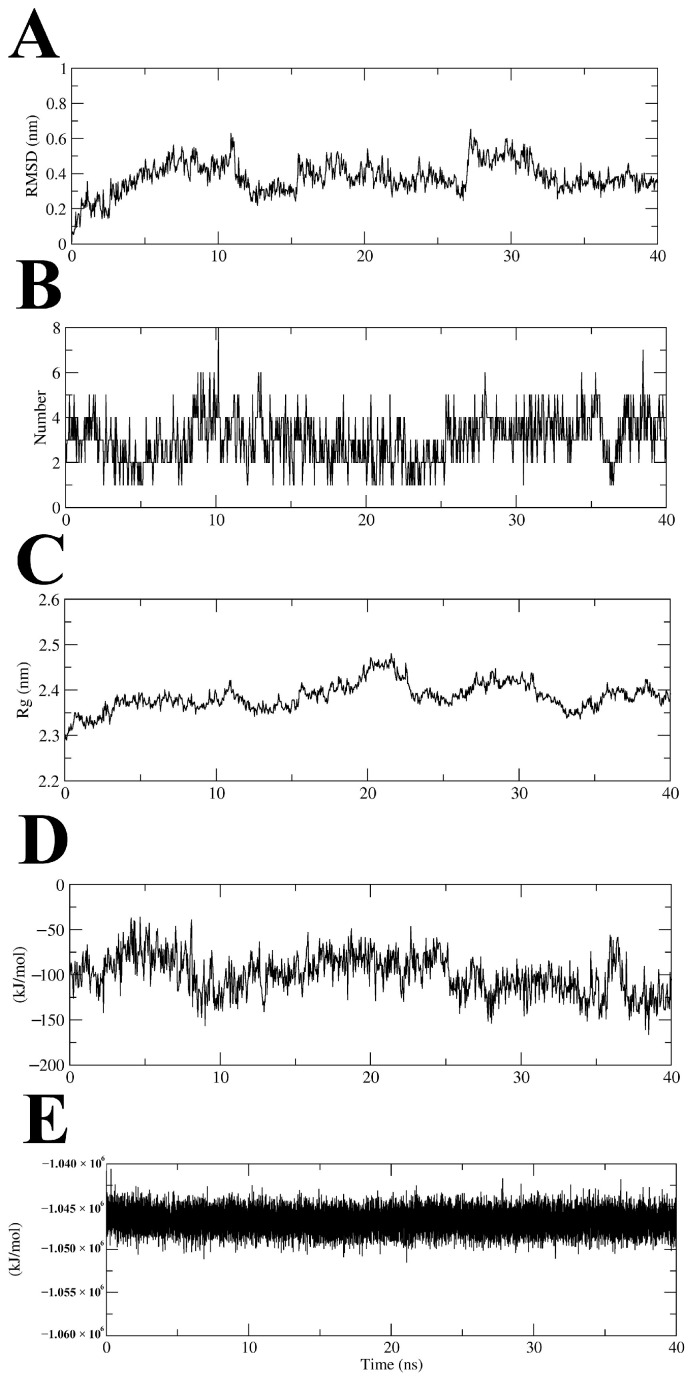
Trajectory analyses of 40 ns MD simulation of MtSAHH–Compound 7 complex. (**A**) Time course change in ligand RMSD value (nm). (**B**) Time course change in hydrogen bond plot. (**C**) Time course change in Rg value (nm). (**D**) Time course change in IE value (kJ/mol). (**E**) Time course change in PE value (kJ/mol).

**Figure 7:**
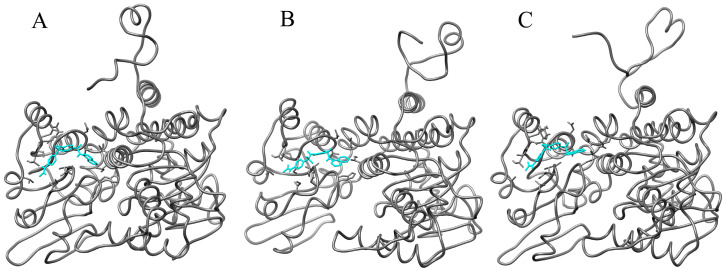
Snapshots of MtSAHH–Compound **7** complex structure in MD simulations. (**A**): 0 ns, (**B**): 20 ns, (**C**): 40 ns. The gray chain indicates MtSAHH structures. Compound **7** is indicated by cyan lines.

**Figure 8:**
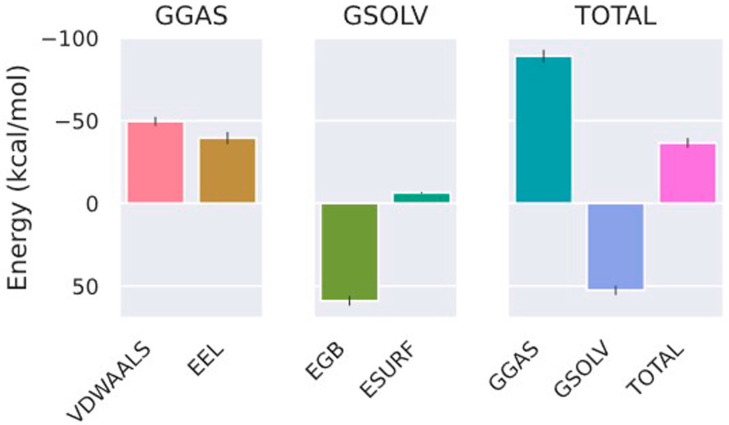
MM/GBSA analysis in MD simulation of MtSAHH–Compound 7 complex. GAS: total gas phase free energy; VDWAALS: van der Waals energy; EEL: electrostatic energy; GSOLV: total solvation free energy; EGB: polar solvation energy; ESURF: nonpolar solvation energy; TOTAL: GGAS + GSOLV.

To evaluate the binding stability of the MtSAHH–Compound **7** complex, the ligand root mean square deviation (RMSD), number of intercomplex hydrogen bonds (Hbonds), complex radius of gyration (Rg), interaction energy (IE), and potential energy (PE) were analyzed using trajectory data from a 40 ns MD simulation. MD simulations in a 40 ns time frame showed that MtSAHH–Compound **7** consistently and stably binds to the active site of MtSAHH, with an average ligand RMSD value of 0.38 nm (Figure 6A). During the MD simulations, two to five (up to eight) hydrogen bonds were constantly formed between Compound **7** and MtSAHH (Figure 6B). The Rg values were constant around 2.3–2.4 nm during MD simulations, indicating that the MtSAHH structure is compact and thus maintains stable folding (Figure 6C). The average value of the IE, which consists of van der Waals interaction and electrostatic interaction, was −99.9 kJ/mol, indicating that Compound **7** stably binds to the active site (Figure 6D). The PE was constant throughout the MD simulation, indicating that the entire system was stable (Figure 6E). Furthermore, a Visual Molecular Dynamics (VMDs) tool was used to visualize the complex structure and confirm the binding during MD simulations. Compound **7** was stably bound to the binding site for 40 ns (Figure 7). The binding free energy of Compound **7** was calculated using the gmx_MMPBSA tool [15,16], and the ΔG_bind_ of Compound **7** was −36.51 kcal/mol (Figure 8). Ligand RMSD and IE values were sufficiently low and stable throughout the simulation period. Hydrogen bonding (the maximum distance between the acceptor and donor atoms was 3.3 Å) was always observed during the simulation period. Furthermore, the bonding free energies by MM/GBSA analysis were low. The evidence indicates that the binding sites of Compound **7** and MtSAHH were always close during the simulation period. The MD simulation results indicated that Compound **7** bound stably to MtSAHH, suggesting that Compound **7** competitively inhibits the enzymatic activity of MtSAHH.

### 2.6. Binding Mode Analysis of MtSAHH–Compound ***7*** Complex

**Figure 9:**
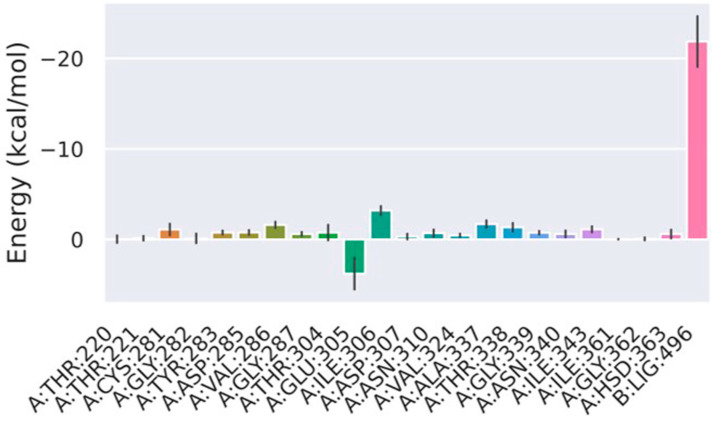
Decomposition analysis of amino acid interaction energy. Decomposition analysis was performed with gmx_MMPBSA tools. Vertical axis: binding free energy values (kcal/mol). Horizontal axis: amino acid residues and numbers.

The interaction mechanism between Compound **7** and MtSAHH was evaluated using a PLIP web server [17]. The naphthylamine group of Compound **7** formed hydrophobic interactions with Thr220, Val286, and His363 and a hydrogen bond with Ala337 (Figure S3). The sulfonyl group of Compound **7** formed hydrogen bonds with Gly284, Asp285, Val286, and Gly287, whereas the phenyl group formed hydrophobic interactions with Thr338 and Ile343 (Figure S3). Among the interacting amino acid residues, Val324 and Ala337 were replaced by Met and Thr in human SAHH, suggesting that Compound **7** would not be able to bind to human SAHH. The non-toxicity of Compound **7** to human HepG2 cells suggests that Compound **7** cannot inhibit the enzymatic activity of human SAHH. The acetamide group of Compound **7** formed hydrophobic interactions with Val324 and Ile343 and hydrogen bonding to Thr304 (Figure S3). The gmx_MMPBSA tools estimated the free energies of each interacting amino acid. The amino acid residues Thr220, Asp285, Val286, Gly287, Val324, Ala337, Thr338, Ile343, and His363 that were predicted by PLIP all showed negative binding free energy (ΔG) values (Figure 9). The relatively high contribution of Val324 and Ala337 to ΔG suggests that amino acid substitutions in humans may reduce the binding ability of Compound **7** to human SAHH. Th recently identified MtSAHH inhibitor forms hydrogen bonds with the 5′ hydroxyl groups of His363 and ADO [18]. His363 has been suggested to act as a switch that opens the gate to allow for SAH binding. Compound **7** also formed a hydrophobic interaction with His363, which likely inhibited the gate-regulatory system.

While the MD simulation and MM/GBSA results suggest that Compound **7** binds to MtSAHH, there is no in vitro or in vivo evidence that Compound **7** binds directly to MtSAHH. Obtaining such evidence will be necessary for future drug discovery using Compound **7** as a lead compound. In recent years, multiple pharmacological properties have been discovered in pharmacologically active compounds, and Compound **7** could also have pharmacological actions targeting proteins other than MtSAHH, although these should be investigated in the future.

### 2.7. ADME/Tox Prediction for Compound **7**

SwissADME [19] was used to predict the pharmacokinetics, drug-likeness, and medicinal chemistry properties of Compound **7**. Bioavailability radar analysis was used to characterize the following six chemical characteristics: saturation, flexibility, solubility, polarity, molecular weight, and lipophilicity [20]. The Lipinski, Veber, and Muegge drug-likeness indices were suitable, whereas Compound **7** deviated from the proper value for the unsaturation parameter (Figure S4). However, its low Gastrointestinal absorption value (GI value) suggests that it may not be suitable for oral administration. ProTox-II [21] was used to predict the toxicity of Compound **7**. Compound **7** was predicted to be non-toxic, with a probability of 0.66 to 0.99 in all 17 toxicity discrimination models (Figure S5). The ProTox-II and SwissADME surveys estimated that Compound **7** is promising for drug development against TB.

## 3. Materials and Methods

### 3.1. Target Protein

The target protein used for SBDS is MtSAHH, and the MtSAHH structure (PDB ID: 3CE6, resolution: 1.60 Å) was obtained from the Research Collaboratory for Structural Bioinformatics Protein Data Bank (RCSB PDB) [22]. The water molecules and the ligand (adenosine) were removed from the crystalline structure. Hydrogen atoms were added to the MtSAHH structure using the Protonate 3D module of the Molecular Operating Environment (MOE) 2019.0102. Subsequently, a partial charge was added to the MtSAHH structure using a partial charge module, and energy minimization was performed using the energy minimize module [23]. The protein structure was optimized using the UCSF chimera [24] and the DOCK program [25] to fit the docking simulation.

### 3.2. Compound Structure Library

The 3D chemical structural library (154,118 chemicals) was obtained from the RPBS Web-based Database [7]. This compound structure library was passed through pharmacokinetic and toxicological evaluation filters for comprehensive safety assessment [26]. In this study, to develop effective and safe drugs, we employed a compound structure library, from which compounds that were unsuitable for pharmaceutical use were eliminated.

### 3.3. Dual Hierarchical In Silico SBDS

The order of the docking simulation tools used in the hierarchical in silico SBDS was examined using EF values. The optimized protein structure processed by MOE was used to perform a molecular surface extraction and pocket search for simulation using DOCK. Molecular surface extraction was performed using the DMS module of the UCSF chimera software, and pocket searches were performed using the sphgen program. We searched for inhibitory compounds in the pocket structure containing the active center of MtSAHH. To use ADV, protein and ligand mol2 files were converted to pdbqt files using autodocktools and searched using ADV’s default search conditions. For the simulation using GOLD, the mol2 files of the protein and ligand were used to calculate the GOLD score (∆$G_{binding}$＝∆$G_{binding}^{0}$ ＋ $E_{clash}$ ＋ $E_{int}$ ＋ $E_{cov}$), with the box center coordinates entered. These three different docking simulation tools (DOCK, ADV, and GOLD) were used to validate the EF_0.2%_. A dataset of DUD-E compounds (544 active compounds and 19,831 inactive compounds) targeting the protein structure of PDBID: 2P54 was used for validation. EF_x%_ was calculated using the following Formula (1):

(1) $${EF}_{x\%}=\frac{\frac{N_{x\%act}}{N_{x\%}}}{\frac{N_{act}}{N_{total}}}$$

N_total_: Total number of compounds;
N_act_: Total number of active compounds;
N_x%_: Number of compounds in the top x% of scores;
N_x%act_: Number of active compounds in the top x% of scores.

### 3.4. Preparation of Compounds

Nine final selected compounds were purchased from ChemBridge, dissolved in 33 mM using DMSO (0.3%) as a solvent, and stored at −80 °C.

### 3.5. Growth Inhibition Assay against Mycobacterium

*M. smegmatis* was obtained from RIKEN, Japan. A total of 3.7% brain–heart infusion broth with pH 7.0 was used for the cultivation of *M. smegmatis*. The culture conditions were as follows: at 37 °C, 240 rpm, for 24 h. The culture medium was then diluted 20-fold. Each compound was adjusted to 33 mM, diluted with culture medium, and added to each well of a 96-well plate by 200 µL. RFP was used as a positive control, and DMSO (0.3%) as a negative control. *M. smegmatis* was incubated in a 96-well plate at 37 °C, 240 rpm, for 24 h. The absorbance of the culture medium was measured at 595 nm using a microplate reader (Bio-Rad Laboratories Inc., Hercules, CA, USA) [27].

### 3.6. Evaluation of Growth Activity against Gram-Negative Bacteria

LB medium with pH 7.0 was used for the cultivation of *E. coli* BL21 strain. The culture conditions were as follows: 37 °C, 240 rpm, for 24 h. The concentration of the compound was adjusted to 100 µM and 200 µL of the culture medium and was added to each well of a 96-well plate. DMSO (0.3%) was used as a negative control, and ABPC was used as a positive control. *E. coli* in a 96-well plate was incubated at 37 °C, 240 rpm, for 8 h. The absorbance of the culture medium was measured at 595 nm after 4 and 8 h using a microplate reader [27].

### 3.7. Toxicity Assays for Human Cells

HepG2 cells were cultured in 96-well plates, and D-MEM medium containing 5% fetal bovine serum, 100 U/mL penicillin, and 100 µg/mL streptomycin was used for cell culture. The culture conditions were follows: at 37 °C in 5% CO_2_ for 3 days. The medium was then replaced with D-MEM medium containing 5% fetal bovine serum, 100 U/mL penicillin, and 100 µg/mL streptomycin. The medium was adjusted to a final concentration of 50 µM of the compound after 24 h and added to each 96-well plate in 150 µL. Cell Counting Kit-8 (Dojin, Kumamoto, Japan) was used to determine the number of viable cells. TCS was used as a positive control, and DMSO (0.3%) was used as a negative control [7]. Cultured cells were incubated for 24 h, and the absorbance of the culture medium was measured at 450 nm using a microplate reader.

### 3.8. MD Simulation

We constructed a simulation system for the complex of Compound **7** and MtSAHH using the CHARMM-GUI web server, Solution Builder [28,29,30]. MD simulations were performed using the GROMACS 2022 [31,32]. The TIP3P was employed as a water model to solubilize a solid box with Compound **7** and the MtSAHH complex. The system was neutralized using Na^+^ and Cl^−^ ion atoms. Neutralizing the system with monovalent cations and monovalent anions is a common technique that is an essential step in the subsequent energy minimization and equilibration [7,33]. A Verlet cutoff scheme of 12 Å was used for electrostatic and van der Waals interactions, and the LINCS algorithm was used for covalent bonding constraints on hydrogen atoms [34]. The particle mesh Ewald method was used to calculate the long-range electrostatic interactions [35]. Energy minimization using the steepest descent method was performed for systems containing 77,598 atoms in 5000 steps. A two-step equilibration was then performed under NVT and NPT conditions, with the temperature and pressure adjusted to 310 K and 1 bar, respectively. Production MD simulations without positional constraints were performed for 40 ns with a time step of 2 fs under NPT conditions. The binding free energy and energy of each interaction residue were calculated using the GB model of the gmx_MMPBSA tool with the last 1 ns of MDS trajectory data. We also used the VMD tool to visualize complex structures and confirm binding during MD simulations.

### 3.9. In Silico Estimation of ADME Properties and Toxicities

In silico estimation of ADME properties and toxicities of compounds were carried out with SwissADME and ProTox-II, respectively.

## 4. Conclusions

Eight compounds with significant inhibitory effects against *Mycobacterium* spp. were identified using novel dual computer-aided drug screening pathways targeting MtSAHH. The prediction rate of the screening pathways was as high as 88.9%, suggesting that this method is extremely effective. Among the hit compounds, the IC_50_ value of Compound **7** was 30.2 μM. Compound **7** was non-toxic to human cells. It also had no growth-inhibitory effects on *E. coli*. MD simulations and the MMPBSA method showed that Compound **7** is stably bound to the active site of MtSAHH. Compound **7** is bound to His363 of MtSAHH, suggesting that it may suppress the gating regulatory system that is essential for substrate binding. Compound **7** has a novel scaffold as a MtSAHH inhibitor, suggesting that it may be a promising new lead compound against *M. tuberculosis*. In the future, further searches for ligand-based analogs of Compound **7** based on key residues would help discover more effective anti-TB drugs.

## Data Availability

The data presented in this study are available upon request from the corresponding author.

## Supplementary Materials

The following supporting information can be downloaded at https://www.mdpi.com/article/10.3390/molecules29061303/s1: Figure S1: Screening pathways and EF values; Figure S2: The active site of MtSAHH; Figure S3: Interaction analysis between Compound **7** and MtSAHH using PLIP; Figure S4: Prediction of pharmacological properties of Compound **7** by SwissADME; Figure S5: Toxicity prediction of Compound **7** by ProTox-II; Table S1: Names, ChemBridgeIDs, IUPAC, and GOLD scores of nine candidate compounds.
