# Supplementary material for: Identification of Novel Antimicrobial Compounds Targeting Mycobacterium tuberculosis S-Adenosyl-L-Homocysteine Hydrolase Using Dual Hierarchical In Silico Structure-Based Drug Screening
Source: Molecules. 2024 Mar 14;29(6):1303. doi: 10.3390/molecules29061303 (PMC10974043; doi:10.3390/molecules29061303)
Supplement: Supplementary file 1 [file molecules-29-01303-s001.zip › molecules-2881130-supplementary.pdf]

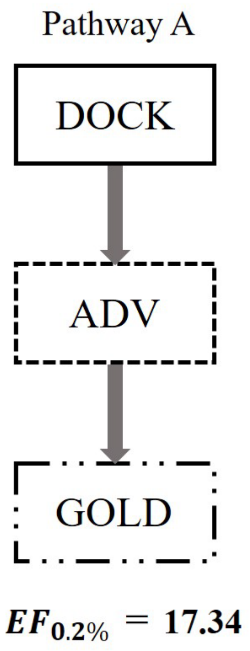

Figure S1. Screening pathways and EF values

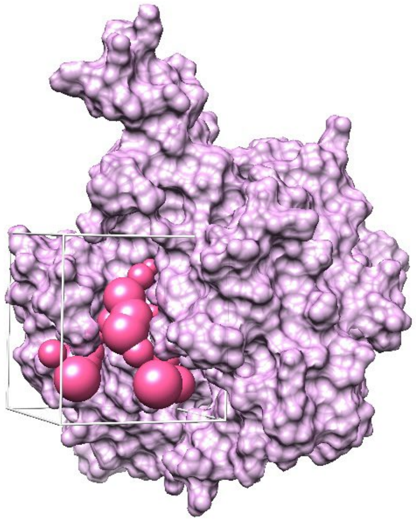

Figure S2. The active site of MtSAHH

Table S1. Names, ChemBridgeIDs, IUPAC names and GOLD scores of nine identified candidate compounds

Name	ChemBridge ID	IUPAC	GOLD score
1	5527036	N-[2-(3,4-dimethoxyphenyl)ethyl]-2-(4-methoxyphenyl)-4-quinolinecarboxamide	80.34[a]<br>82.81[b]
2	6531848	N-(4-{[4-(3-phenoxybenzyl)-1-piperazinyl]sulfonyl}phenyl)acetamide oxalate	82.03[a]<br>82.12[b]
3	6240894	4-methoxy-N-{4-[4-(4-morpholinylsulfonyl)phenyl]-1,3-thiazol-2-yl}benzamide	80.40[a]<br>79.70[b]
4	6238996	N-{4-[4-(4-morpholinylsulfonyl)phenyl]-1,3-thiazol-2-yl}-2-phenoxyacetamide	83.16[a]
5	7280375	N-[4-(aminosulfonyl)benzyl]-2-(4-bromophenyl)-4-quinolinecarboxamide	80.37[a]
6	7921920	N-{2-[4-(aminosulfonyl)phenyl]ethyl}-2-(3-isopropoxyphenyl)-4-quinolinecarboxamide	80.02[a]
7	6720847	N-{4-[({4-[(1-naphthylamino)sulfonyl]phenyl}amino)sulfonyl]phenyl}acetamide	82.51[b]
8	7432424	2-{[3-(2,3-dihydro-1,4-benzodioxin-2-ylmethyl)-4-oxo-3,4-dihydro-2-quinazolinyl]thio}-N-(3-methoxyphenyl)acetamide	81.95[b]
9	7923531	4-{[4-(3-methoxybenzyl)-1-piperazinyl]carbonyl}-6-methyl-2-(4-pyridinyl)quinoline	80.18[b]

Note: a; pathway A, b; pathway C.

LIG (AC1L7OZ3)

LIG-Z-0

Interacting chains: D

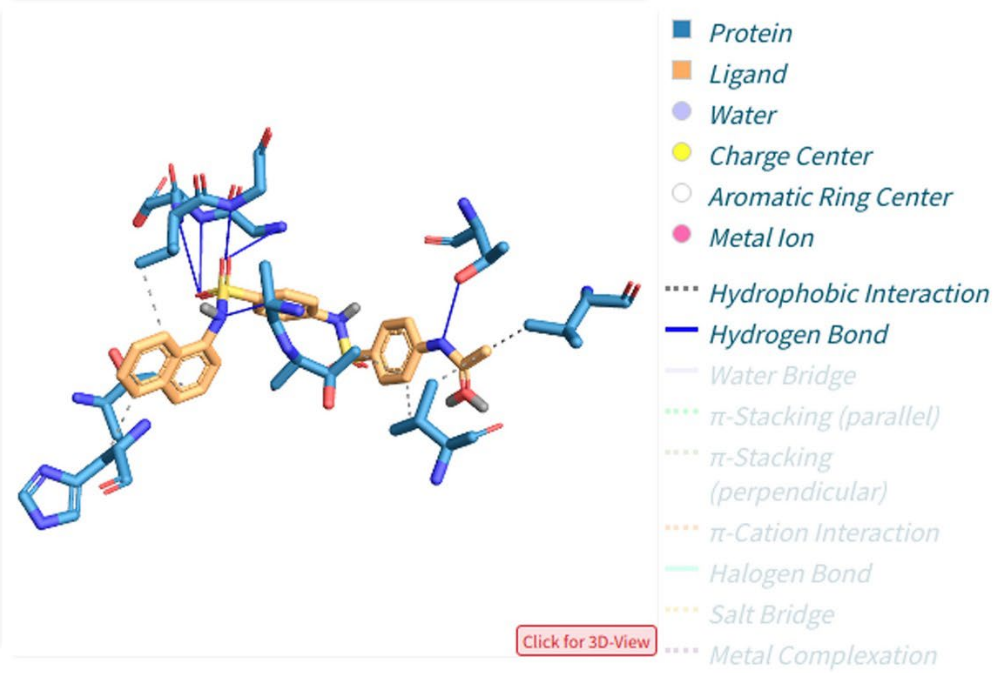

Hydrophobic Interactions

Index	Residue	AA	Distance	Ligand Atom	Protein Atom
1	220D	THR	3.61	7449	3246
2	286D	VAL	3.32	7453	4237
3	324D	VAL	3.51	7474	4779
4	338D	THR	3.31	7467	4970
5	343D	ILE	3.63	7474	5039
6	343D	ILE	3.55	7467	5040
7	363D	HIS	3.68	7456	5357

Hydrogen Bonds

Index	Residue	AA	Distance H-A	Distance D-A	Donor Angle	Protein donor?	Side chain	Donor Atom	Acceptor Atom
1	284D	GLY	3.02	3.51	111.24	✓	×	4212 [Nam]	7478 [O-]
2	285D	ASP	3.28	3.69	105.82	✓	×	4219 [Nam]	7477 [O-]
3	286D	VAL	2.81	3.80	167.34	✓	×	4231 [Nam]	7477 [O-]
4	287D	GLY	1.75	2.71	157.11	✓	×	4247 [Nam]	7478 [O-]
5	304D	THR	2.11	3.09	163.07	×	✓	7471 [Nam]	4481 [O3]
6	337D	ALA	1.47	2.40	148.48	×	×	7446 [Npl]	4957 [O2]

Figure S3. Interaction analysis between Compound 7 and MtSAHH using PLIP

Molecule 1

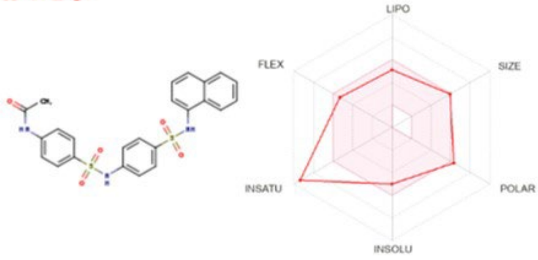

SMILES [CH2]C(=O)Nc1ccc(cc1)S(=O)(=O)Nc1ccc(cc1)S(=O)(=O)Nc1cccc2c1cccc2

Physicochemical Properties	
Formula	C24H20N3O5S2
Molecular weight	494.56 g/mol
Num. heavy atoms	34
Num. arom. heavy atoms	22
Fraction Csp3	0.04
Num. rotatable bonds	8
Num. H-bond acceptors	5
Num. H-bond donors	3
Molar Refractivity	131.43
TPSA	138.20 Å²

Lipophilicity	
Log $P_{o/w}$ (iLOGP)	0.00
Log $P_{o/w}$ (XLOGP3)	3.40
Log $P_{o/w}$ (WLOGP)	5.80
Log $P_{o/w}$ (MLOGP)	2.38
Log $P_{o/w}$ (SILICOS-IT)	1.84
Consensus Log $P_{o/w}$	2.68

Water Solubility	
Log *S* (ESOL)	-5.00
Solubility	4.96e-03 mg/ml ; 1.00e-05 mol/l
Class	Moderately soluble
Log *S* (Ali)	-5.98
Solubility	5.16e-04 mg/ml ; 1.04e-06 mol/l
Class	Moderately soluble
Log *S* (SILICOS-IT)	-9.28
Solubility	2.63e-07 mg/ml ; 5.31e-10 mol/l
Class	Poorly soluble

Pharmacokinetics	
GI absorption	Low
BBB permeant	No
P-gp substrate	No
CYP1A2 inhibitor	No
CYP2C19 inhibitor	Yes
CYP2C9 inhibitor	Yes
CYP2D6 inhibitor	No
CYP3A4 inhibitor	Yes
Log K_p (skin permeation)	-6.90 cm/s

Druglikeness	
Lipinski	Yes; 0 violation
Ghose	No; 3 violations: MW>480, WLOGP>5.6, MR>130
Veber	Yes
Egan	No; 1 violation: TPSA>131.6
Muegge	Yes
Bioavailability Score	0.55

Medicinal Chemistry	
PAINS	0 alert
Brenk	0 alert
Leadlikeness	No; 2 violations: MW>350, Rotors>7
Synthetic accessibility	2.96

Figure S4. Prediction of pharmacological properties of Compound **7** by SwissADME

Toxicity Model Report

Classification	Target	Shorthand	Prediction	Probability
Organ toxicity	Hepatotoxicity	dili	Inactive	0.66
Toxicity end points	Carcinogenicity	carcino	Inactive	0.69
Toxicity end points	Immunotoxicity	immuno	Inactive	0.99
Toxicity end points	Mutagenicity	mutagen	Inactive	0.81
Toxicity end points	Cytotoxicity	cyto	Inactive	0.83
Tox21-Nuclear receptor signalling pathways	Aryl hydrocarbon Receptor (AhR)	nr_ahr	Inactive	0.93
Tox21-Nuclear receptor signalling pathways	Androgen Receptor (AR)	nr_ar	Inactive	0.99
Tox21-Nuclear receptor signalling pathways	Androgen Receptor Ligand Binding Domain (AR-LBD)	nr_ar_lbd	Inactive	0.99
Tox21-Nuclear receptor signalling pathways	Aromatase	nr_aromatase	Inactive	0.98
Tox21-Nuclear receptor signalling pathways	Estrogen Receptor Alpha (ER)	nr_er	Inactive	0.94
Tox21-Nuclear receptor signalling pathways	Estrogen Receptor Ligand Binding Domain (ER-LBD)	nr_er_lbd	Inactive	0.99
Tox21-Nuclear receptor signalling pathways	Peroxisome Proliferator Activated Receptor Gamma (PPAR-Gamma)	nr_ppar_gamma	Inactive	0.97
Tox21-Stress response pathways	Nuclear factor (erythroid-derived 2)-like 2/antioxidant responsive element (nrf2/ARE)	sr_are	Inactive	0.98
Tox21-Stress response pathways	Heat shock factor response element (HSE)	sr_hse	Inactive	0.98
Tox21-Stress response pathways	Mitochondrial Membrane Potential (MMP)	sr_mmp	Inactive	0.71
Tox21-Stress response pathways	Phosphoprotein (Tumor Supressor) p53	sr_p53	Inactive	0.94
Tox21-Stress response pathways	ATPase family AAA domain-containing protein 5 (ATAD5)	sr_atad5	Inactive	0.99

Figure S5. Toxicity prediction of Compound **7** by ProTox-II
